# Self-monitoring of Physical Activity After Hospital Discharge in Patients Who Have Undergone Gastrointestinal or Lung Cancer Surgery: Mixed Methods Feasibility Study

**DOI:** 10.2196/35694

**Published:** 2022-06-24

**Authors:** Marijke Elizabeth de Leeuwerk, Martine Botjes, Vincent van Vliet, Edwin Geleijn, Vincent de Groot, Erwin van Wegen, Marike van der Schaaf, Jurriaan Tuynman, Chris Dickhoff, Marike van der Leeden

**Affiliations:** 1 Rehabilitation Medicine Amsterdam University Medical Centers location Vrije Universiteit Amsterdam Amsterdam Netherlands; 2 Ageing & Vitality Amsterdam Movement Sciences Amsterdam Netherlands; 3 Rehabilitation & Development Amsterdam Movement Sciences Amsterdam Netherlands; 4 Rehabilitation Medicine Amsterdam University Medical Centers location University of Amsterdam Amsterdam Netherlands; 5 Center of Expertise Urban Vitality Faculty of Health Amsterdam University of Applied Sciences Amsterdam Netherlands; 6 General Surgery Amsterdam University Medical Centers location Vrije Universiteit Amsterdam Amsterdam Netherlands; 7 Cancer Centre Amsterdam, Treatment and Quality of Life Amsterdam Netherlands; 8 Cardio-Thoracic Surgery Amsterdam University Medical Centers location Vrije Universiteit Amsterdam Amsterdam Netherlands

**Keywords:** mobile phone, physical activity, self-monitoring, fitness trackers, telemedicine, cancer, physical therapy

## Abstract

**Background:**

Self-monitoring of physical activity (PA) using an accelerometer is a promising intervention to stimulate PA after hospital discharge.

**Objective:**

This study aimed to evaluate the feasibility of PA self-monitoring after discharge in patients who have undergone gastrointestinal or lung cancer surgery.

**Methods:**

A mixed methods study was conducted in which 41 patients with cancer scheduled for lobectomy, esophageal resection, or hyperthermic intraperitoneal chemotherapy were included. Preoperatively, patients received an ankle-worn accelerometer and the corresponding mobile health app to familiarize themselves with its use. The use was continued for up to 6 weeks after surgery. Feasibility criteria related to the study procedures, the System Usability Scale, and user experiences were established. In addition, 6 patients were selected to participate in semistructured interviews.

**Results:**

The percentage of patients willing to participate in the study (68/90, 76%) and the final participation rate (57/90, 63%) were considered good. The retention rate was acceptable (41/57, 72%), whereas the rate of missing accelerometer data was relatively high (31%). The mean System Usability Scale score was good (77.3). Interviewed patients mentioned that the accelerometer and app were easy to use, motivated them to be more physically active, and provided postdischarge support. The technical shortcomings and comfort of the ankle straps should be improved.

**Conclusions:**

Self-monitoring of PA after discharge appears to be feasible based on good system usability and predominantly positive user experiences in patients with cancer after lobectomy, esophageal resection, or hyperthermic intraperitoneal chemotherapy. Solving technical problems and improving the comfort of the ankle strap may reduce the number of dropouts and missing data in clinical use and follow-up studies.

## Introduction

Surgery is an essential curative treatment option for patients diagnosed with gastrointestinal or lung cancer; however, it has a major impact on daily functioning and quality of life [1-3]. Most patients experience incomplete or delayed recovery of physical functioning after major thoracic or abdominal surgery [2-4].

During hospitalization, stimulation of physical activity (PA) has been shown to enhance the recovery of physical functioning [2,4-6], reduce the postoperative risk of readmission, and shorten the length of hospital stay [7,8]. Therefore, PA promotion is integrated into Enhanced Recovery After Surgery (ERAS) programs [9]. The aim of ERAS programs is to reduce postoperative complications and improve postoperative recovery. However, ERAS programs are mainly limited to the period of hospitalization, whereas encouraging PA after hospital discharge is also important for improving functional recovery [10,11].

In their own environments, increasing PA levels and resuming daily activities can be challenging for patients. They may experience barriers such as physical symptoms, insecurity, lack of motivation, or social support in doing so [12]. The use of body-worn accelerometers can support patients in resuming their daily activities after cancer surgery [10,13]. Such devices enable self-monitoring of and feedback on PAs by quantifying the frequency and intensity of human movement [14].

Adequate use of accelerometers for PA self-monitoring is an important prerequisite for its potential positive effect on functional recovery. Several studies have shown the feasibility of PA self-monitoring in patients who have undergone major (oncological) surgery, each using a different device [15,16]. Qualitative data on experiences with PA self-monitoring in these patients are largely unknown [17], and these experiences may add to the knowledge about potential barriers to the use of this technology and may help resolve them [18].

Therefore, this study aimed to collect both quantitative and qualitative data to investigate the feasibility of self-monitoring of PA using accelerometers after hospital discharge in patients with cancer who have undergone gastrointestinal or lung cancer surgery.

## Methods

### Ethics Approval

The study was approved by the medical research ethics committee of the Amsterdam University Medical Centers, location Vrije Universiteit Medical Center (registration number 2018/112). All patients provided written informed consent.

### Study Design

A feasibility study with a mixed methods design was performed between April 2019 and April 2020 in patients with gastrointestinal or lung cancer scheduled for surgery. The formal sample size was not calculated. Instead, a convenience sample with a 1-year inclusion period was chosen. Self-monitoring of PA after hospital discharge was evaluated using a questionnaire and interviews conducted in April 2020, and the study procedures were evaluated using administrative data during the course of the study.

### Participants

The inclusion criteria were adult patients with gastrointestinal or lung cancer who were invited for preoperative physiotherapy screening between April 2019 and April 2020 at the outpatient clinic of our tertiary teaching hospital (Amsterdam University Medical Center, location Vrije Universiteit Medical Center), which included patients scheduled for a lobectomy, esophageal resection, or hyperthermic intraperitoneal chemotherapy (HIPEC). Exclusion criteria were <7 days between inclusion and surgery, emergency procedures, patients who are nonambulatory, and no access to or not able to use a smartphone or tablet.

### Intervention

**Figure 1:**
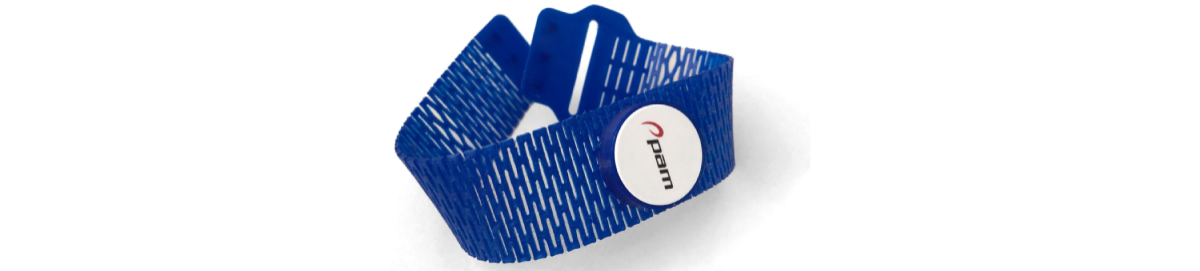
The Physical Activity Monitor.

**Figure 2:**
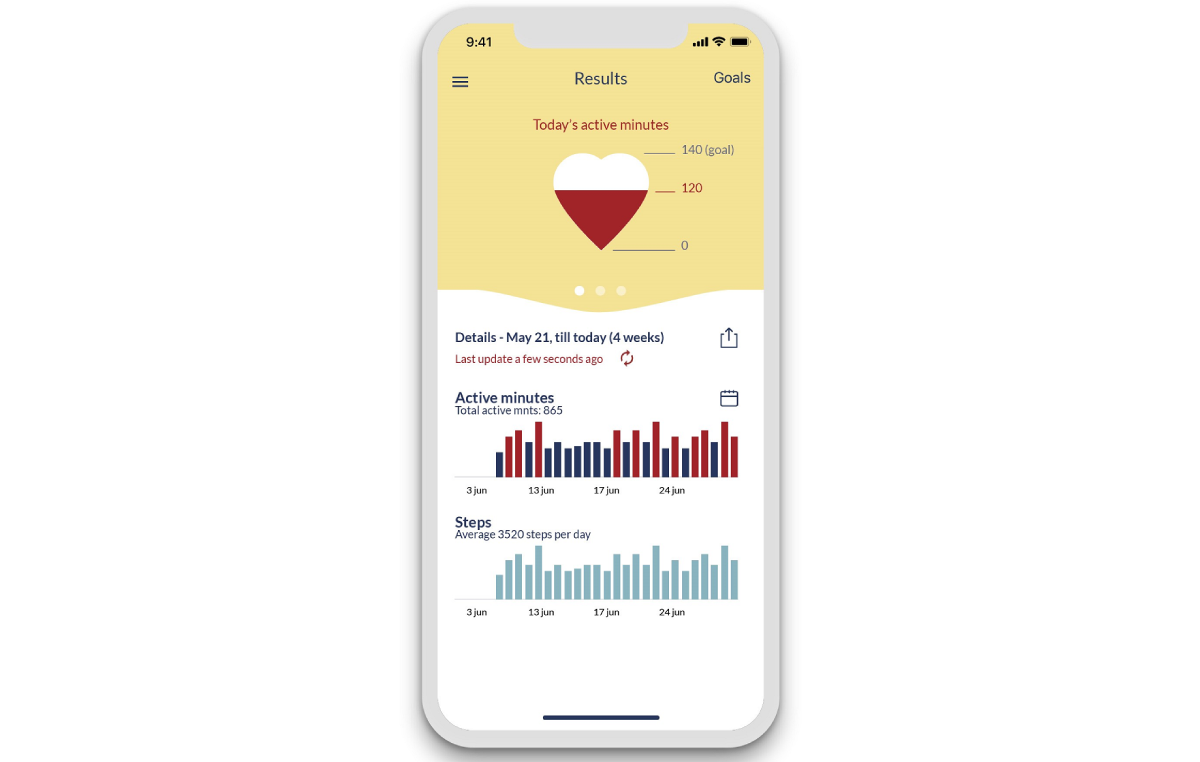
Atris app.

Potentially eligible patients were informed about the study by the treating physiotherapist during the preoperative consultation. Patients who were willing and eligible to participate received the Physical Activity Monitor (PAM) AM400 3-axis accelerometer (Pam BV Doorwerth) and were given access to the corresponding smartphone app called *Atris* (Peercode BV, Geldermalsen; Figures 1 and 2). The PAM was selected for this study as (1) the PAM AM400 was found to be a suitable movement sensor to validly measure activity minutes [19]; (2) the battery of the PAM lasts for approximately 1 year, eliminating the need for patients to recharge the device; (3) the data of the PAM can be synced directly to a web-based application, enabling remote monitoring by clinicians; and (4) the PAM can be worn around the ankle and is waterproof to allow 24/7 wearing. The PAM measures PA continuously and provides the total PA every 15 minutes. With the *Atris app*, patients were able to self-monitor their daily PA levels and received feedback on the number of active minutes per day. Patients were able to set personal activity goals in the app by themselves.

All patients received the usual pre- and postoperative physiotherapy care. During the standard preoperative consultation, potential risk factors (eg, smoking or sedentary lifestyle) for delayed postoperative recovery were identified, leading to personalized advice for improving preoperative physical fitness. In addition, the patients were given instructions about the PAM and *Atris* app. The physiotherapist informed the patient that the app provided insights into the recovery of PA and advised them to use the app to resume their daily activities after surgery. Patients were asked to start wearing the PAM 24 hours a day in a strap around the ankle for at least 7 days before surgery to familiarize themselves with its use.

During hospitalization, patients received standard physiotherapy consultations and were stimulated to mobilize according to a daily mobilization goal following the ERAS protocol [20]. In addition, the physiotherapist guided the patients in using the PAM and *Atris* app.

Personalized rehabilitation recommendations were provided at discharge. If indicated by the physiotherapist, the patients were advised to continue physiotherapy in primary care after discharge. For this study, the patients were asked to wear the PAM 24 hours a day for 6 weeks. A period of 6 weeks was chosen as it was expected that patients would be able to gain sufficient experience using the PAM and *Atris* app to assess feasibility. A hospital physiotherapist provided insights into the activity levels of the patient on the corresponding web application and monitored the activity data of the patients weekly. In case the activity levels decreased or no data were available, the physiotherapist contacted the patient. In the case of technical problems, the physiotherapist helped resolve them if possible. In cases where patients had problems resuming their PA level by themselves, the physiotherapist advised them to contact a physiotherapist in primary care. The patients also had the opportunity to contact the physiotherapist themselves.

### Outcome Measures

#### Study Procedures

Patients who were willing to participate (out of potentially eligible patients invited to the study), those who ultimately participated, and those who completed the study were recorded and described as percentages. Furthermore, the amount, type, and reasons for missing PAM data were identified. For exploration purposes, the PAM data were described as the number of active minutes per day (24 hours) from 1 week before surgery (baseline) to 6 weeks after surgery.

#### System Usability

System usability was assessed using the Dutch translation of the System Usability Scale (SUS). The SUS contains 10 statements about efficiency, learnability, and satisfaction and has been validated to assess the usability of electronic systems [21]. Patients can indicate the degree of agreement with each statement on a 5-point Likert scale. The total SUS score ranges from 0 to 100, with a score of ≥70 considered good. The patients received an email in April 2020 to complete the questionnaire on a secured web-based system (Castor Electronic Data Capture).

#### User Experiences

In addition to the SUS, the patients received 13 additional questions about the acceptability, satisfaction, and added value of the PAM and *Atris* app that was experienced (see Multimedia Appendix 1 for the questionnaire). In addition, the user experiences of the patients were assessed using semistructured qualitative interviews (see Multimedia Appendix 2 for the topic list). The responses to the SUS and additional questions were used as supplemental topics to the topic list. All patients were asked if they were willing to participate in the interviews. There were 2 groups, patients who did and patients who did not experience additional value. Of both groups, 3 patients were randomly selected for the interview. Interviews were conducted by VvV and MEdL via telephone and recorded using a voice recorder. Interviews were transcribed verbatim.

#### Descriptive Data

Demographic and clinical data were collected retrospectively from electronic medical records.

### Feasibility Criteria

To evaluate the feasibility of self-monitoring of PA using the PAM and *Atris* app, we set feasibility criteria a priori based on cutoff points described in previous studies (Textbox 1) [21-25].

To better understand the feasibility, additional qualitative data on acceptability, satisfaction, and experienced additional value were collected to explore user experiences.

**Feasibility criteria based on cutoff points described in previous studies.**

**Willingness to participate**

- Percentage of invited, potentially eligible, patients who were willing to participate in the study; a percentage of >70% was considered feasible

**Participation rate**

- Percentage of willing and eligible patients who intended to participate in the study; a percentage of >60% was considered feasible

**Retention rate**

- Percentage of included patients who completed the study (ie, these patients did not explicitly indicate their decision to stop); a retention rate of 80% was considered feasible

**Data collection**

- Percentage of missing Physical Activity Monitor data was determined to investigate whether physical activity data collection using the Physical Activity Monitor was feasible; complete data in at least 70% of all participants were considered feasible

**System usability**

- Measured with the System Usability Scale; a score of ≥70 was considered good

### Data Analysis

SPSS (version 26; IBM Corp) was used for quantitative data analysis. Study population characteristics were presented descriptively as mean (SD), median (IQR), and percentage. Quantitative data were analyzed descriptively. The study procedures were presented as percentages. PAM data were considered missing if they were not available for ≥3 days in a given week. The available PAM data are presented as the median (IQR) of the total active minutes per day of each week and as a percentage of the preoperative PA baseline level. The mean (SD) SUS was calculated using the method described in the study by Brooke [21]. Additional questions about user experience are presented as percentages.

The research software ATLAS.ti (version 8) was used for qualitative analysis. Qualitative data analysis was performed following the steps of thematic analysis by 2 researchers (MvdL and MB) [26]. The interviews were read several times to familiarize with the data. Data were open coded line by line to segment them into the initial codes. Axial coding was used to define the definitive codes. Definitive codes were classified and described under different themes [26].

## Results

### Overview

**Figure 3:**
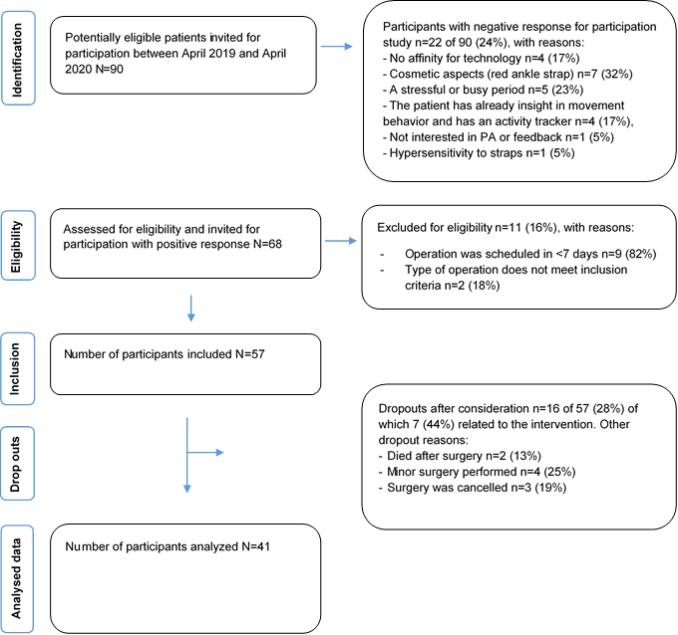
Flow of participants through the study. PA: physical activity.

A total of 90 potentially eligible patients were invited to participate between April 2019 and April 2020, of whom 68 (76%) were willing to participate. After the final eligibility check, of the 90 patients, 57 (63%) were included in the study, resulting in a participation rate of 63%. The retention rate was 72% (41/57); 28% (16/57) of patients dropped out during the study, of whom 44% (7/16) were related to the intervention. The reasons for nonparticipation, exclusion, and dropout are presented in the flowchart (Figure 3). Ultimately, 41 patients were included in the analysis. The median age of the patients in this study was 68 (IQR 60-73) years, and 58% (25/41) were male. The most common types of surgery were lobectomy (23/41, 56%) and HIPEC (10/41, 24%). The median length of hospital stay was 7 (IQR 6-11) days. Other relevant demographic and clinical data are presented in Table 1.

**Figure 4:**
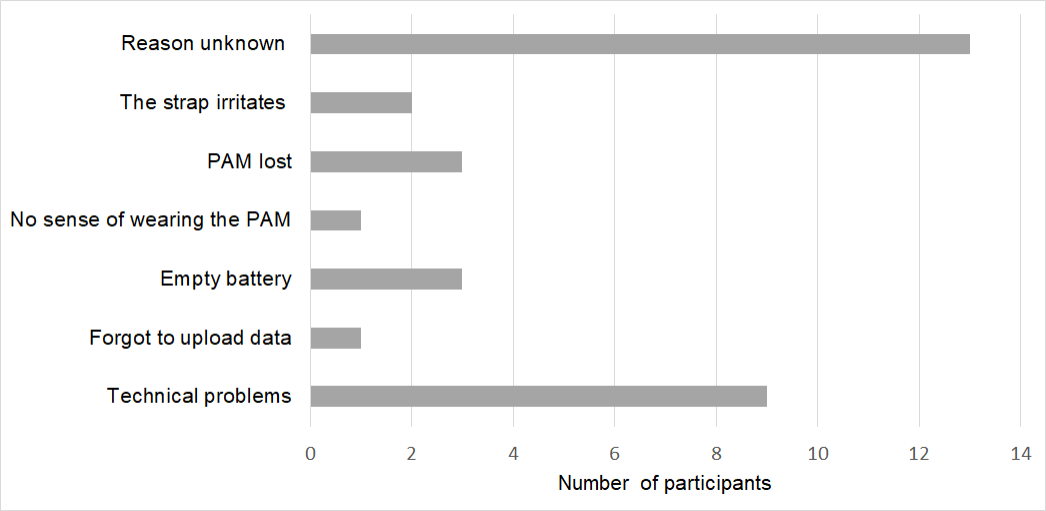
Reasons for missing data. PAM: Physical Activity Monitor.

Approximately, 31% of the PAM data were missing. Missing value analysis suggested that the data were missing at random, as missing data increased during the time blocks of the postoperative phase. The amount of missing data increased during the 6 postoperative weeks: 27% of the PAM data were missing in the first postoperative week and 44% in the sixth postoperative week. Figure 4 shows an overview of the reasons for the missing data. The most common reasons for missing data were technical problems or withdrawal of wearing the PAM. Table 2 shows the median preoperative and postoperative PA levels in minutes per day and the median percentage of recovery in PA compared with the preoperative levels.

**Table 1:** Patient characteristics (N=41).

Variable				Results
**Patient characteristics**				
	Sex (male), n (%)			25 (58)
	Age (years), median (IQR)			68 (60-73)
	BMI, mean (SD)			26.1 (4.2)
	**Smoke status, n (%)**			
		Current	5 (12)	
		Past	24 (59)	
		Never	12 (29)	
	**Primary diagnosis, n (%)**			
		Rectum cancer	1 (2)	
		Lung cancer	21 (51)	
		Esophagus cancer	8 (20)	
		Peritonitis carcinomatosa	8 (20)	
		Schwannoma	2 (5)	
		Thymoma	1 (2)	
	**Tumor stage, n (%)**			
		1	7 (17)	
		2	8 (20)	
		3	6 (15)	
		4	18 (44)	
		Schwannoma	2 (5)	
	**Comorbidities (ASA^a^ score), n (%)**			
		Grade I	3 (7)	
		Grade II	27 (66)	
		Grade III	10 (24)	
		Grade IV	1 (2)	
	**Type of treatment before surgery, n (%)**			
		Neoadjuvant chemotherapy	3 (7)	
		Neoadjuvant chemoradiotherapy	9 (22)	
		Neoadjuvant immunotherapy	1 (2)	
		Neoadjuvant hormone therapy	1 (2)	
	**Sports ≥1 time per week, n (%)**			15 (37)
		Missing	1 (2)	
**Perioperative characteristics**				
	**Type of surgery, n (%)**			
		Lobectomy	23 (56)	
		Esophagus resection	7 (17)	
		HIPEC^b^ procedure	10 (24)	
		Schwannoma resection	1 (2)	
	**Surgical approach, n (%)**			
		Video-assisted thoracic surgery	6 (15)	
		Open surgery	35 (85)	
	**Type of treatment after surgery, n (%)**			
		Adjuvant chemoradiotherapy	2 (5)	
		Adjuvant chemotherapy	3 (7)	
		Adjuvant radiotherapy	1 (2)	
		Adjuvant hormonotherapy	1 (2)	
	Length of stay (days), median (IQR)			7 (6-11)
	**Complications (Clavien Dindo score), n (%)**			
		Grade I	26 (63)	
		Grade II	5 (12)	
		Grade IIIa	2 (5)	
		Grade IIIb	5 (12)	
		Grade IVa	3 (7)	
	Hospital readmission, n (%)			5 (12)
	Duration of operation (minutes), median (IQR)			200 (128-336)

^a^ASA: American Society of Anesthesiologists.

^b^HIPEC: hyperthermic intraperitoneal chemotherapy.

**Table 2:** Median PA^a^ (minutes per week) and percentage of PA compared with preoperative levels of PA (N=41)^b^.

PA level			Preoperative		1 week^c^		2 weeks		3 weeks		4 weeks		5 weeks		6 weeks
**PA (minutes)**															
	Values, median (IQR)	172 (114-213)		51 (26-82)		87 (54-138.5)		96 (68.5-171.5)		108 (78.3-170.5)		118.5 (80-196.5)		139 (81-184)	
	Values, n (%)	38 (93)		30 (73)		29 (71)		29 (71)		28 (68)		28 (68)		27 (66)	
**PA compared with preoperative level of PA** **(%)**															
	Values, median (IQR)	N/A^d^		29.4 (17.9-47.4)		55.7 (34.1-77.0)		65.0 (44.9-84.3)		67.2 (52.1-93.5)		78.0 (49.6-101.9)		80.3 (57.6-99.7)	
	Values, n (%)	N/A		29 (71)		27 (66)		26 (63)		25 (61)		25 (61)		25 (61)	

^a^PA: physical activity.

^b^PA at baseline (preoperative; time point 0) and 1 to 6 weeks postoperative (time point 1 to time point 6).

^c^After surgery.

^d^N/A: not applicable.

### Feasibility

#### Overview

The results of the feasibility criteria are presented in Table 3.

**Table 3:** Summary of results of feasibility criteria.

Feasibility criteria		Targets	Results	Conclusions
**Study procedures**				
	Willingness to participate	Percentage of willing patients >70%	76% of the invited patients were willing to participate	Feasible
	Participation rate	Participation rate >60%	The participation rate was 63%	Feasible
	Retention rate	A retention rate of >80%	The number of dropouts during the study was 16; this resulted in a retention rate of 72%	Marginally feasible
	Data collection	Complete outcome data of PA^a^ in at least 70% of all participants at follow-up	Approximately 31% of the PA data were missing; the number of complete cases was 9, and 8 cases had <10% missing data	Not feasible
**System usability**				
	Efficiency	SUS^b^ score ≥70	Mean 79.6 (SD 24.2)	Feasible
	Learnability	SUS score ≥70	Mean 74.0 (SD 27.5)	Feasible
	Satisfaction	SUS score ≥70	Mean 75.0 (SD 25.2)	Feasible
**User experience**				
	Study patients	Qualitative data about acceptability, satisfaction, and experienced added value	Wearing the PAM^c^ was acceptable, patients were largely positive about the PAM and Atris app, and most patients experienced an added value; technical problems and the comfort of the ankle strap need to be improved	Feasible

^a^PA: physical activity.

^b^SUS: System Usability Score.

^c^PAM: Physical Activity Monitor.

#### SUS and Additional Questions

Of the 41 patients, the SUS and additional questionnaires were sent to 39 (95%) patients (n=2, 5% of patients died before April 2020). The mean number of weeks between the end of the self-monitoring period and receiving the questionnaire was 21.6 (SD 17.0). Approximately 85% (33/39) of patients responded to the questionnaire, of whom 5% (2/39) did not complete the entire questionnaire. System usability was feasible, with a mean SUS score of 77.3 (SD 20.7). Of all responding patients, 75% would recommend other patients to use the PAM and *Atris* app after surgery. Most patients (84%) indicated that they wore the PAM all day during the study period. The reasons for not wearing the PAM were poor comfort with the ankle strap or technical problems (eg, connection problems between the PAM and app). The other outcomes of the questionnaire are presented in Multimedia Appendix 3.

#### Interviews

#### Overview

**Figure 5:**
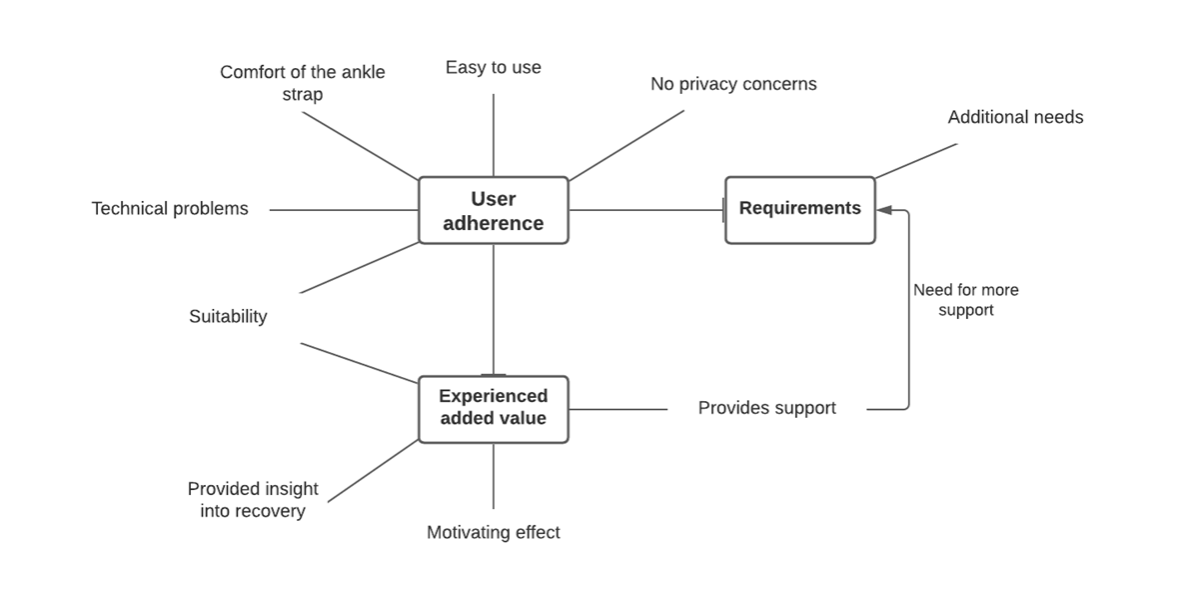
Code tree.

Of the 39 patients, 8 (21%) patients did not respond to the interview invitation, and 4 (10%) were unwilling to participate in additional interviews. Of the remaining 27 patients who were willing to participate, 8 (30%) patients did not, and 19 (70%) patients found that the use of the PAM and *Atris* app added value. Approximately 15% (6/39) of patients were selected. The characteristics of the interviewed patients are shown in Table 4. The results are described by themes in the following paragraphs and supported by quotes (Table 5). The code tree is shown in Figure 5.

**Table 4:** Characteristics of interviewed patients.

Interviewee number	Gender	Age (years)	Type of surgery	ASA^a^	Length of hospital stay (days)	Missing data (%)	SUS^b^ score
1	Male	69	HIPEC^c^ (open)	1	11	69.4 (reason unknown)	70
2	Female	61	Lobectomy (VATS^d^)	2	3	63.3 (PAM^e^ lost)	90
3	Male	66	Lobectomy (VATS)	2	11	0	85
4	Male	77	Lobectomy (VATS)	2	4	34 (connection lost between PAM and smartphone)	97.5
5	Male	56	Lobectomy (open)	2	7	34.7 (no connection between PAM and smartphone)	67.5
6	Male	73	Lobectomy (open)	2	5	26.5 (low battery)	92.5

^a^ASA: American Society of Anesthesiologists.

^b^SUS: System Usability Scale.

^c^HIPEC: hyperthermic intraperitoneal chemotherapy.

^d^VATS: video-assisted thoracic surgery.

^e^PAM: Physical Activity Monitor.

**Table 5:** Quotations of interviewed participants.

Quotation		Code	Interviewed participant number
**User adherence**			
	“By the way, that bracelet was awful. Especially the closure. You have to invest a bit more in that. I don’t know much else to improve. It weights nothing. You even forget it once in a while.” (quote 1)	Comfort of the ankle strap	2
	“Every morning and during the day I had to put the bracelet back on again, because it would be loose for a while, but that wasn’t that bad...I don’t have any complaints about it, that bracelet is a simple but good solution for wearing the sensor.” (quote 2)	Comfort of the ankle strap	6
	“In the beginning I had some trouble with updating. The connection wasn’t always good. I have a certain brand of phone and apparently it doesn’t work as well as other phones. Later I did a new update and then it worked better. I also had some contact with the VUmc about this.” (quote 3)	Technical problems	4
	“It didn’t work well at all times. Then I called for a new battery. Then it worked again.” (quote 4)	Technical problems	5
	“And again about the technical problems. That really frustrated me. I called with the VUmc for help. They could often improve it remotely and the new battery helped in the end.” (quote 5)	Technical problems	5
	“I found the use of the app very friendly. Very easy, absolutely not unnecessarily complicated.” (quote 6)	Easy to use	2
	“It was a new experience for me. But I had no problems at all with it. It all went well.” (quote 7)	Easy to use	6
	“I have not thought for a moment of not using it because of my privacy. Only my active minutes were registered and I did not see any reason not to use it.” (quote 8)	No privacy concerns	6
**Experienced added value**			
	“I thought it was a phenominal item...You keep track of your active minutes in the app during the day. I do not use cell phones very often, because I am 63 years old, but this was a very nice challenge.” (quote 9)	Provided insights into recovery	2
	“I found it a nice application, I watched it every day.” (quote 10)	Provided insights into recovery	6
	“It had really became part of my lifestyle. When I went to sleep I took it off and put it on the bedside table. Before I took a shower I put it o, so every active minute would count. I had the feeling that the health professionals from the Vumc did everything they could, so I wanted to do that myself. This device helped me a lot with that.” (quote 11)	Provided support	5
	“Well if we look at the operation, especially my recovery, then it’s very important to me that I have insight in and influence on my recovery. That you are able to see if you’re making progress. When I just started I was 30 minutes active per day and at the end I was 4 to 5 hours active. It is very nice and important to have that insight.” (quote 12)	Provided insights into recovery	3
	“Well it worked really stimulating for me. Making movement goals gives direction in the rehabilitation proces. You can work towards that. It has really helped me and that’s why I would recommend it to others.” (quote 13)	Motivating effect	5
	“You feel more co-responsible. Well then it’s nice that you can show you are dowing well and that you try your best.” (quote 14)	Motivating effect	3
	“It’s an addition to you health and life. It makes rehabilitation a little easier and more challenging. It focuses more on recovery than on your problems.” (quote 15)	Provided support	2
	“It’s all very frightening and scary. What is going to happen? Will I wake up after the operation? Can I still do the same as I did? There is a lot going through you head and it’s pretty scary, to be honest. At that moment, health profesionnals and such a motion sensor around you ankle helps enormously. You get feedback and it gives you something to hold on to.” (quote 16)	Provided support	5
	“I think it is a very good remedy. Only for myself it had not been necessary. For someone who has more difficulty with being active this is a completely different story. Then it can be a very nice support. If I speak for myself, it was just that I was curious about how much I walked that day.” (quote 17)	Suitability	4
	“I already moved a lot: I go to the gym twice a week and I also walk a lot and cycle a bit. Therefore, the PAM wasn’t the reason I started being more active. But it was nice to see how active I was during the day.” (quote 18)	Suitability	6
**Requirements**			
	“I would like to get a signal if I don’t show good or abnormal activity behavior. Starting a conversation. That’s also possible by telephone. I don’t necessarily have to come to the VUmc more often. But such a conversation would be very nice.” (quote 19)	Need for more support	1
	“A sort of alarm or stimulating message. I think that also helps in creating awareness. People need to become aware of their activity behavior. A message when things are not going that well can help with that. It triggers you to think about it.” (quote 20)	Need for more support	2
	“I would like to get some more information as well. So besides the activity data. For example, about the heartrate and blood pressure. But anyway, it might also be difficult to integrate that into one application.” (quote 21)	Additional needs	4
	“Activity data should be more clearly displayed. Now you have a graph, but the activity data is only presented per day. You actually want to be able to see data and differences during the day. For example my difference in activity between an evening shift and day shift at work. I would have found that interesting to be able to see.” (quote 22)	Additional needs	5
	“I think it might work better if the goals are better tailored to the person.” (quote 22)	Additional needs	5

#### User Adherence

Topics that may have had a negative impact on user adherence included problems experienced with the ankle straps and technical problems. All the interviewed patients experienced problems with the closure of the strap. They mentioned occasional loosening of the strap, as the closure did not function properly (quote 1; Table 5). Despite this problem, wearing the bracelet was an obstacle for none of the patients (quote 2; Table 5). In total, 67% (4/6) of patients mentioned technical problems; sometimes the connection between the sensor and the app did not work, disabling the update of the activity data (quote 3; Table 5). Of these 4 patients, 2 (50%) received a new sensor as the battery was depleted prematurely (quote 4; Table 5). One of the participants mentioned that the technical problems were frustrating (quote 5; Table 5).

Topics that may have had a positive effect on user adherence were ease of use of the *Atris* app (quotes 6 and 7; Table 5) and absence of privacy concerns (quote 8; Table 5).

#### Experienced Added Value

Most of the interviewed patients were positive about the use of the PAM and *Atris* app (quotes 9 and 10; Table 5). One of the participants mentioned that the use of the PAM had become a part of his lifestyle (quote 11; Table 5). All patients experienced having more insight into their recovery with the use of the PAM and *Atris* app as they were able to see if they were making progress (quote 12; Table 5). In addition, they mentioned that the PAM and *Atris* app had a motivating effect. They stimulated them to be more physically active as they were able to set goals and they felt more coresponsible for their recovery (quotes 13 and 14; Table 5). Moreover, patients experienced the PAM and *Atris* app as support during their recovery process. They mentioned that they provided more focus on recovery and provided something to hold on to (quotes 15 and 16; Table 5).

Approximately 33% (2/6) of patients did not experience additional values but were positive about the concept. They mentioned that they were already motivated to be physically active regardless of the PAM. They thought it would be more suitable for patients who needed more motivation to be physically active (quotes 17 and 18; Table 5).

#### Requirements

Overall, 67% (4/6) of patients highlighted the need for more support. They mentioned that it would be of additional value if they received messages or calls in situations of insufficient or abnormal activity behavior (quote 19; Table 5). Moreover, 33% (2/6) of patients also mentioned that motivational messages might serve as additional incentives (quote 20, Table 5). Additional requirements mentioned by 4 patients were the possibility to add additional measurements of data, such as heart rate or blood pressure (quote 21; Table 5). In addition, 33% (2/6) of patients wanted to gain more insight into the activity pattern during the day (quote 22; Table 5); 33% (2/6) of patients highlighted the need for more personalization (quote 23; Table 5).

## Discussion

### Principal Findings

Self-monitoring of PA after discharge appears to be feasible based on good system usability and predominantly positive user experiences in patients with cancer after lobectomy, esophageal resection, or HIPEC. These findings are consistent with those of other studies [15-17,22]. Wu et al [15] found good feasibility of self-monitoring using a wrist-worn accelerometer and an app in patients after gastric cancer surgery. Low et al [17] reported good usability of a real-time mobile technology–based sedentary behavior intervention for patients with abdominal cancer in the perioperative period using a smartwatch. However, feasibility was considered moderate in that study as adherence to wearing the smartwatch decreased significantly from before to after the surgery. In our study, adherence also seemed to decrease based on an increase in missing data during the intervention period. Solving technical problems and improving the comfort of the ankle strap may reduce the number of dropouts and missing data in clinical use and follow-up studies. In addition, improving self-efficacy and self-motivation and engaging in more social support could enhance user adherence, as suggested in a systematic review of predictors of adherence in home-based physical rehabilitation [27].

The system usability of the PAM and *Atris* app is similar to that of devices used in other studies [17,25]. Jonker et al [25] reported good system usability (mean SUS 73.1) for a wrist-worn activity tracker and mobile app in older adult patients after oncological surgery. In a study by Low et al [17], the system usability of a Fitbit smartwatch with an accompanying smartphone app during the perioperative period in patients scheduled for abdominal cancer surgery was also found to be good (mean SUS 83.8). The qualitative data, in addition to the quantitative data, provided insights into the facilitators of and barriers to the use of the *Atris* app and PAM. The user experiences were largely positive. The interviewed patients mentioned that the PAM and *Atris* app were easy to use, motivated them to be more physically active, and provided support after discharge. However, most of the patients recommended the design of a more comfortable ankle strap, and some were annoyed about technical problems. Only a few patients did not experience the added value of the PAM and *Atris* app as, in their opinion, they were already sufficiently active and, therefore, did not feel the need for additional support. In contrast, some other patients indicated the need for more support, such as through occasional telephone contact with a physiotherapist or motivational messages. Therefore, the tailoring of interventions to individual needs and preferences should be considered.

In this study, we explored the course of recovery in PA in the first 6 weeks after surgery. These results may be supportive in clinical practice to gain more objective insights into patient recovery and identify which patients may need more support in improving their PA levels. We found that most (19/25, 76%) of our study population did not return to baseline PA levels 6 weeks after surgery, although these results should be interpreted with caution, given the relatively large amount of missing PA data. In previous observational pilot studies using objective PA data after (cancer) surgery, most patients did not reach preoperative PA levels even at 3 months after surgery [28,29]. Similarly, a study using questionnaires to investigate the course of recovery in physical functioning 6 and 12 weeks after lung cancer surgery showed that patients were still recovering between 6 and 12 weeks after surgery [4]. The patients in our study underwent major surgeries, including HIPEC and major lung resections. These procedures are both associated with prolonged functional recovery compared with less invasive procedures such as minimally invasive segmental colectomies or video-assisted small lung resections. Therefore, it is suggested that for most of these patients, the period of supportive care to improve PA should be >6 weeks after surgery. However, to increase user adherence for longer-term use, the previously mentioned improvements to the ankle bracelet and resolution of technical problems are necessary. In addition, to better understand all dimensions of user adherence, an in-depth analysis of adherence to ambulant monitoring in this patient population should be performed, taking into account the 5 dimensions of adherence as described by the World Health Organization [30].

By conducting this feasibility study, barriers and enablers were identified for the use of the PAM and *Atris* app after hospital discharge in patients after cancer surgery. However, proper technical functioning and comfort in wearing are important prerequisites for all activity trackers. Moreover, the enablers found in our study, for example, that it motivates patients to be more physically active and that it provides more insight into PA recovery, are also generalizable to other activity trackers. The cost-effectiveness and effectiveness of interventions using PA self-monitoring during cancer treatment is largely unknown, and the conduct of randomized clinical trials is warranted [31]. In addition, not all patients seem to require the same amount of postoperative support. Further research should take into account the risk of functional decline after surgery, as well as the needs and preferences of individual patients.

### Limitations

This study had some limitations. First, this feasibility study was conducted in a single hospital setting. In addition, not all diagnoses within gastrointestinal and lung cancer surgery were represented in our study population as preoperative physiotherapy screening was not part of the care pathway for all patients in our hospital. Therefore, the results of this study cannot be generalized to other diagnoses. Second, all patients were contacted to ask whether they were willing to participate in an interview after PA data collection had already ended. However, some patients did not respond or were unwilling to participate in the interviews. This could have caused a selection bias. To reduce selection bias among the patients interviewed, they were selected based on whether they perceived the PAM and *Atris* app as adding value. However, the selected interviewees had a somewhat higher mean SUS score than that of the entire study population (83.8 vs 77.3). Thus, this approach probably did not sufficiently eliminate the selection bias. To gain a full understanding of feasibility, future studies should also interview nonparticipants. In addition, data saturation may not have been achieved as the interviews were conducted in a small and partly selective sample. Third, for some patients, there was a period of several months between the end of the self-monitoring period and the completion of the questionnaires (and interviews), which may have led to recall bias. Finally, as the study was conducted in the usual care setting, the perioperative instructions from the physiotherapist were not strictly followed as per protocol, which hindered reproducibility. Our research group is currently working on a protocol to guide physiotherapists in using this intervention.

### Conclusions

The results of our study showed good system usability and predominantly positive user experiences in patients with cancer after lobectomy, esophageal resection, or HIPEC. Most patients mentioned that the PAM and *Atris* app motivated them to be more physically active after discharge. The retention rate and amount of missing data need to be improved in follow-up studies. Solving technical problems and improving the comfort of ankle straps may enhance user adherence, thereby reducing the number of dropouts and missing data. Randomized clinical trials should be conducted to investigate whether interventions using accelerometers indeed improve the recovery of PA and physical functioning after surgery in this population.

## Appendix

Multimedia Appendix 1 Additional feasibility questions.

Multimedia Appendix 2 Topic list interviews.

Multimedia Appendix 3 Outcomes feasibility questionnaire.

## Abbreviations

ERAS: Enhanced Recovery After Surgery
HIPEC: hyperthermic intraperitoneal chemotherapy
PA: physical activity
PAM: Physical Activity Monitor
SUS: System Usability Scale
