# Supplementary material for: Self-monitoring of Physical Activity After Hospital Discharge in Patients Who Have Undergone Gastrointestinal or Lung Cancer Surgery: Mixed Methods Feasibility Study
Source: JMIR Cancer. 2022 Jun 24;8(2):e35694. doi: 10.2196/35694 (PMC9270713; doi:10.2196/35694)
Supplement: Multimedia Appendix 1 [file cancer_v8i2e35694_app1.docx]

**Supplementary file 1:** Additional feasibility questions

De onderstaande stellingen gaan over het gebruikt van de Atris app. Geef bij elke stelling aan in welke mate u het eens bent met de stelling 1= helemaal niet mee eens, 5 = helemaal mee eens.

*The statements below are about using the Atris app. For each statement, indicate to what extent you agree with the statement 1 = totally disagree, 5 = totally agree*

**Vraag 1:** De PAM en Atris app hebben mij gemotiveerd tot meer bewegen.

***Question 1:*** *The PAM and Atris app have motivated me to move more.*

**Vraag 2:** De PAM en Atris app hebben bijgedragen aan mijn lichamelijke herstel.

***Question 2:*** *The PAM and Atris app have contributed to my physical recovery.*

**Vraag 3:** De PAM en Atris app waren een toegevoegde waarde op mijn revalidatieproces.

***Question 3:*** *The PAM and Atris app were an added value to my rehabilitation process.*

**Vraag 4:** Door de Atris app ben ik mij meer bewust geworden van mijn beweeggedrag.

***Question 4:*** *Because of the Atris app I have become more aware of my movement behavior.*

**Vraag 5:** Ik had hulp nodig om te kunnen starten met de PAM en Atris app.

***Question 5****: I needed help to get started with the PAM and Atris app.*

**Vraag 6:** Mijn telefoon werkte goed bij het gebruik van de Atris app.

***Question 6:*** *My phone worked well when using the Atris app*

**Vraag 7:** Ik zou andere patiënten aanraden om de PAM en Atris app te gebruiken als zij geopereerd moeten worden.

***Question 7****: I would advise other patients to use the PAM and Atris app if they need surgery.*

**Vraag 8:** Er zijn twee soorten enkelbandjes gebruikt gedurende de onderzoeksperiode. Welk enkelbandje had u gekregen: het bandje met een gesp of het bandje zonder een gesp?

- Bandje met gesp
- Bandje zonder gesp
- Weet ik niet meer

***Question 8:*** *Two types of ankle straps were used during the research period. Which ankle strap did you get: the strap with a clasp or the strap without a clasp?*

- *Strap with clasp*
- *Strap without clasp*
- *I don't remember*

**Vraag 9:** Ik vond het enkelbandje van de PAM comfortabel om te dragen.

***Question 9****: I found the PAM ankle bracelet comfortable to wear.*

**Vraag 10:** Op hoeveel dagen per week heeft u de PAM gedragen?

- Nooit
- Minder dan 2 dagen per week
- 2-4 dagen per week
- 5-7 dagen per week
- Alle dagen

***Question 10:*** *On how many days a week did you wear the PAM?*

- *Never*
- *Less than 2 days a week*
- *2-4 days a week*
- *5-7 days a week*
- *All days*

**Vraag 10_1:** Wat was de reden dat u de PAM niet droeg?

- Ik was het vergeten
- De PAM zat niet comfortabel
- Er waren technische problemen met de PAM/app
- Ik vond het enkelbandje niet mooit
- Anders

***Question 10_1****: What was the reason you were not wearing the PAM?*

- *I had forgotten*
- *The PAM was not comfortable*
- *There were technical problems with the PAM/app*
- *I didn't like the ankle bracelet*
- *Otherwise*

**Vraag 10_1_1 :** Licht hieronder uw antwoord toe. (Indien antwoord op de vorige vraag was 5 ‘Anders’)

***Question 10_1_1 :*** *Please explain your answer below. (If the answer to the previous question was 5 'Otherwise')*

**Vraag 11:** Hoe vaak kreeg u direct contact met de sensor als u de Atris app opende?

- Nooit
- Nauwelijks
- Soms
- Vaak
- Altijd

***Question 11:*** *How often did you get direct contact with the sensor when you opened the Atris app?*

- *Never*
- *Hardly*
- *Sometimes*
- *Often*
- *Always*

**Vraag 12:** Hoe vaak keek u gemiddeld in de Atris app om uw activiteit in te zien?

- Nooit
- Minder dan 1x per week
- 1x per week
- Om de dag
- 1-3 keer per dag
- Vaker dan 3x per dag

***Question 12:*** *How often on average did you look in the Atris app to see your activity?*

- *Never*
- *Less than 1x per week*
- *1x per week*
- *Every other day*
- *1-3 times a day*
- *More often than 3 times a day*

**Vraag 13:** Als u de keuze zou hebben, hoe zou u dan graag begeleid willen worden in het opbouwen van uw activiteiten tijdens uw revalidatie?

- Ik wil liever door niemand begeleid worden
- Face to face contact met een fysiotherapeut
- Berichtjes via de Atris app
- Telefonisch door de fysioterhapeut
- Anders

***Question 13:*** *If you had the choice, how would you like to be guided in building up your activities during your rehabilitation?*

- *I would rather not be guided by anyone*
- *Face to face contact with a physiotherapist*
- *Messages via the Atris app*
- *Telephone call with a physiotherapist*
- *Otherwise*

**Vraag 13_1:** Licht hieronder uw antwoord toe. (Indien antwoord op vraag 23 was ‘Anders’)

***Question 13_1:*** *Explain your answer below. (If answer to question 23 was 'Otherwise’)*
