# Supplementary material for: Self-monitoring of Physical Activity After Hospital Discharge in Patients Who Have Undergone Gastrointestinal or Lung Cancer Surgery: Mixed Methods Feasibility Study
Source: JMIR Cancer. 2022 Jun 24;8(2):e35694. doi: 10.2196/35694 (PMC9270713; doi:10.2196/35694)
Supplement: Multimedia Appendix 2 [file cancer_v8i2e35694_app2.docx]

**Supplementary file 2:** Topic list interviews

- Algemene ervaring met de PAM & Atris app
- In hoeverre aansluiting bij wensen en behoeften
- PAM en betrokkenheid patiënt
- PAM in de thuissituatie
- PAM in het ziekenhuis
- PAM en praktisch
- PAM en de techniek
- Beperkingen/onbenutte mogelijkheden PAM
- Toekomst van de PAM

*English:*

*- General experience with the PAM & Atris app*

*- The extent to which they fit in with wishes and needs*

*- PAM and patient involvement*

*- PAM in the home situation*

*- PAM in the hospital*

*- PAM and practical*

*- PAM and the technique*

*- Restrictions/unutilized PAM capabilities*

*- Future of the PAM*
