# Supplementary material for: Self-monitoring of Physical Activity After Hospital Discharge in Patients Who Have Undergone Gastrointestinal or Lung Cancer Surgery: Mixed Methods Feasibility Study
Source: JMIR Cancer. 2022 Jun 24;8(2):e35694. doi: 10.2196/35694 (PMC9270713; doi:10.2196/35694)
Supplement: Multimedia Appendix 3 [file cancer_v8i2e35694_app3.docx]

**Supplementary file 3:** outcomes feasibility questions

***Question 1:*** *The PAM and Atris app have motivated me to move more. (1 = totally disagree, 5 = totally agree)*

| **N=33** | **N (%)** |
| --- | --- |
| 1 | 8 (24,2) |
| 2 | 2 (6,1) |
| 3 | 2 (6,1) |
| 4 | 6 (18,2) |
| 5 | 15 (45,5) |

***Question 2:*** *The PAM and Atris app have contributed to my physical recovery. (1 = totally disagree, 5 = totally agree)*

| **N=33** | **N (%)** |
| --- | --- |
| 1 | 8 (24,2) |
| 2 | 1 (3,0) |
| 3 | 2 (6,1) |
| 4 | 6 (18,2) |
| 5 | 16 (48,5) |

***Question 3:*** *The PAM and Atris app were an added value to my rehabilitation process. (1 = totally disagree, 5 = totally agree)*

| **N=33** | **N (%)** |
| --- | --- |
| 1 | 8 (24,2) |
| 2 | 0 (0) |
| 3 | 4 (12,1) |
| 4 | 5 (15,2) |
| 5 | 16 (48,5) |

***Question 4:*** *Because of the Atris app I have become more aware of my movement behavior. (1 = totally disagree, 5 = totally agree)*

| **N=33** | **N (%)** |
| --- | --- |
| 1 | 5 (15,2) |
| 2 | 1 (3,0) |
| 3 | 4 (12,1) |
| 4 | 9 (27,3) |
| 5 | 14 (42,4) |

***Question 5****: I needed help to get started with the PAM and Atris app. (1 = totally disagree, 5 = totally agree)*

| **N = 32** | **N (%)** |
| --- | --- |
| 1 | 18 (56,3) |
| 2 | 3 (9,4) |
| 3 | 1 (3,1) |
| 4 | 6 (18,8) |
| 5 | 4 (12,5) |

***Question 6:*** *My phone worked well when using the Atris app. (1 = totally disagree, 5 = totally agree)*

| **N = 32** | **N (%)** |
| --- | --- |
| 1 | 5 (15,6) |
| 2 | 2 (6,3) |
| 3 | 5 (15,6) |
| 4 | 6 (18,8) |
| 5 | 14 (43,8) |

***Question 7****: I would advise other patients to use the PAM and Atris app if they need surgery. (1 = totally disagree, 5 = totally agree)*

| **N = 32** | **N (%)** |
| --- | --- |
| 1 = helemaal niet mee eens | 5 (15,6) |
| 2 | 0 (0) |
| 3 | 3 (9,4) |
| 4 | 6 (18,8) |
| 5 = helemaal mee eens | 18 (56,3) |

***Question 8:*** *Two types of ankle straps were used during the research period. Which ankle strap did you get: the strap with a clasp or the strap without a clasp?*

| **N = 32** | **N (%)** |
| --- | --- |
| I don’t remember | 4 (12,5) |
| Strap without clasp | 18 (56,3) |
| Strap with clasp | 10 (31,3) |

***Question 9****: I found the PAM ankle bracelet comfortable to wear. (1 = totally disagree, 5 = totally agree)*

| **N = 32** | **N (%)** |
| --- | --- |
| 1 | 5 (15,6) |
| 2 | 5 (15,6) |
| 3 | 9 (28,1) |
| 4 | 6 (18,8) |
| 5 | 7 (21,9) |

***Question 10:*** *On how many days a week did you wear the PAM?*

| **N =32** | **N (%)** |
| --- | --- |
| Never | 0 (0) |
| Less than 2 days a week | 2 (6,3) |
| 2-4 days a week | 1 (3,1) |
| 5-7 days a week | 2 (6,3) |
| All days | 27 (84,4) |

***Question 10_1****: What was the reason you were not wearing the PAM?*

| **N = 5** | **N (%)** |
| --- | --- |
| I had forgotten | 0 (0) |
| The PAM was not comfortable | 2 (40,0) |
| There were technical probles with the PAM/app | 1 (20,0) |
| I didn’t like the ankle bracelet | 0 (0) |
| Otherwise | 2 (40,0) |

***Question 10_1_1 :*** *Please explain your answer below. (If the answer to the previous question was 5 'Otherwise') (N=2)*

*- At the latest after 7 days of trying to open on the phone, the app did not emer. Just as good friends, well used and ready. Sent back again.*

*- Sat well.*

***Question 11:*** *How often did you get direct contact with the sensor when you opened the Atris app?*

| **N = 32** | **N (%)** |
| --- | --- |
| Never | 3 (9,4) |
| Hardly | 2 (6,3) |
| Sometimes | 4 (12,5) |
| Often | 17 (53,1) |
| Always | 6 (18,8) |

***Question 12:*** *How often on average did you look in the Atris app to see your activity?*

| **N = 32** | **N (%)** |
| --- | --- |
| Never | 2 (6,3) |
| Less than 1x per week | 0 (0) |
| 1x per week | 3 (9,4) |
| Every other day | 7 (21,9) |
| 1-3 times a day | 13 (40,6) |
| More often than 3 times a day | 7 (21,9) |

***Question 13:*** *If you had the choice, how would you like to be guided in building up your activities during your rehabilitation?*

| **Antwoord** | **N (%)** |
| --- | --- |
| I would rather not be guided by anyone | 2 (6,3) |
| Face to face contact with a physiotherapist | 7 (21,9) |
| Messages via the Atris app | 13 (40,6) |
| Telephone call with a physiotherapist | 10 (31,3) |
| Otherwise | 0 (0) |

***Question 13_1:*** *Explain your answer below. (If answer to question 23 was 'Otherwise’) (N=0)*
